# Should we care about *Plasmodium vivax* and HIV co-infection? A systematic review and a cases series from the Brazilian Amazon

**DOI:** 10.1186/s12936-020-03518-9

**Published:** 2021-01-06

**Authors:** Paola López Del-Tejo, Nadia Cubas-Vega, Cecilia Caraballo-Guerra, Bernardo Maia da Silva, Jefferson da Silva Valente, Vanderson Souza Sampaio, Djane Clarys Baia-da-Silva, Daniel Barros Castro, Flor Ernestina Martinez-Espinosa, André Machado Siqueira, Marcus Vinícius Guimarães Lacerda, Wuelton Marcelo Monteiro, Fernando Val

**Affiliations:** 1 Programa de Pós-Graduação Em Medicina Tropical, Universidade do Estado do Amazonas, Manaus, Brazil; 2 Fundação de Medicina Tropical Dr. Heitor Vieira Dourado, Manaus, Brazil; 3 Fundação de Vigilância em Saúde do Amazonas, Manaus, Brazil; 4 Programa de Pós-Graduação em Ciências da Saúde, Universidade Federal Do Amazonas, Manaus, Brazil; 5 Instituto Leônidas and Maria Deane, FIOCRUZ, Manaus, Brazil; 6 Instituto Nacional de Infectologia Evandro Chagas, FIOCRUZ, Rio de Janeiro, Brazil

**Keywords:** *Plasmodium vivax*, HIV, Co-infection, Systematic review, Epidemiology

## Abstract

**Background:**

Malaria and HIV are two important public health issues. However, evidence on HIV-*Plasmodium vivax* co-infection (HIV/PvCo) is scarce, with most of the available information related to *Plasmodium falciparum* on the African continent. It is unclear whether HIV can change the clinical course of vivax malaria and increase the risk of complications. In this study, a systematic review of HIV/PvCo studies was performed, and recent cases from the Brazilian Amazon were included.

**Methods:**

Medical records from a tertiary care centre in the Western Brazilian Amazon (2009–2018) were reviewed to identify HIV/PvCo hospitalized patients. Demographic, clinical and laboratory characteristics and outcomes are reported. Also, a systematic review of published studies on HIV/PvCo was conducted. Metadata, number of HIV/PvCo cases, demographic, clinical, and outcome data were extracted.

**Results:**

A total of 1,048 vivax malaria patients were hospitalized in the 10-year period; 21 (2.0%) were HIV/PvCo cases, of which 9 (42.9%) had AIDS-defining illnesses. This was the first malaria episode in 11 (52.4%) patients. Seven (33.3%) patients were unaware of their HIV status and were diagnosed on hospitalization. Severe malaria was diagnosed in 5 (23.8%) patients. One patient died. The systematic review search provided 17 articles (12 cross-sectional or longitudinal studies and 5 case report studies). A higher prevalence of studies involved cases in African and Asian countries (35.3 and 29.4%, respectively), and the prevalence of reported co-infections ranged from 0.1 to 60%.

**Conclusion:**

Reports of HIV/PvCo are scarce in the literature, with only a few studies describing clinical and laboratory outcomes. Systematic screening for both co-infections is not routinely performed, and therefore the real prevalence of HIV/PvCo is unknown. This study showed a low prevalence of HIV/PvCo despite the high prevalence of malaria and HIV locally. Even though relatively small, this is the largest case series to describe HIV/PvCo.

## Background

Malaria and human immunodeficiency virus (HIV) infections are two major public health concerns [1]. Given the considerable epidemiological overlap between malaria and HIV, a substantial number of co-infections may occur [1]. In 2019, malaria accounted for 228 million cases and resulted in 405,000 deaths, globally [2]. Of the 5 *Plasmodium* species that can infect humans, *Plasmodium vivax* is the most geographically widespread, and is responsible for 75% of malaria-related cases in the Latin American region [2–4]. Currently, 37.9 million people are living with HIV worldwide, with 1.7 million newly diagnosed in 2019. An estimated 690,000 deaths were due to AIDS-related diseases in 2019 [5]. Globally, these infections jointly claim the lives of about 2 million people each year [6].

Malaria and HIV infections can interact in a bidirectional and synergistic manner, which may lead to an exponential rise in their deleterious effects [7]. HIV can impair immune responses to malaria parasites, and lead to an inability to control parasite clearance, thus resulting in high parasitic loads, which in turn, can increase malaria transmission rates [1, 8, 9]. Clinically, HIV has been shown to contribute to a higher incidence of falciparum malaria [10], including its severe form, which is characterized by anaemia, cerebral malaria and increased risk of congenital infections [11–13]. The impact of HIV on the severity of malaria appears to be restricted to patients with CD4 + T cell counts < 350 cells/μL [10].

Meanwhile, malaria infection is associated with strong CD4 + T cell activation and increased levels of pro-inflammatory cytokines, which provides an ideal micro-environment for HIV viral replication. This potentially worsens the clinical picture, increasing HIV progression to AIDS and maintaining the viral cycle [1, 14–18]. The immunosuppression caused by HIV infection can reduce the control of the *Plasmodium* infection. Moreover, HIV therapy can impair malaria treatment, with a significant increase in adverse events, as well as potential selection of treatment-resistant parasites [19–23]. *Plasmodium* co-infection has also been shown to increase HIV viral load and transiently decrease CD4 + T cell count [24–27]. However, these interactions are mostly described for *Plasmodium falciparum.*

Studies reporting HIV-*P. vivax* co-infection (HIV/PvCo) are scarce. Therefore, a clear understanding of the interaction between these two diseases is necessary for more effective control measures, especially in co-endemic areas of vivax malaria and HIV. In this study, clinical and laboratory outcomes in a case series of HIV/PvCo patients admitted to a tertiary care centre in the Western Brazilian Amazon is described. Also, evidence regarding HIV/PvCo in the literature is provided through a systematic review of published articles.

## Methods

### Case series

All medical records from patients admitted to the Fundação de Medicina Tropical Dr Heitor Vieira Dourado (FMT-HVD) for suspected vivax malaria infection, from March 2009 to December 2018, were screened for eligibility for this study. The FMT-HVD is a tertiary reference health care centre for infectious diseases in Manaus, Western Brazilian Amazon and receives patients directly seeking care and those referred by public and private care networks in Manaus and the surrounding cities. Around 30% of all malaria cases in the municipality are diagnosed in the FMT-HVD and 90% of people living with HIV are followed up at this health care unit. Manaus is the capital of the Amazonas state, has a total area of 11,401 sq km and a population of 2.2 million habitants (population density of 194.7 hab/sq km). The FMT-HVD is part of the Brazilian free public health system (*Sistema Único de Saúde*—SUS) and adopts all Brazilian guidelines for the management of sexually transmitted infections [28], including HIV infection in adults [29], as well as malaria treatment [30].

All patients are registered in the hospital’s electronic medical records (EMR). Data used in this study were obtained after a thorough examination of the hospital’s database. All information regarding patient demographics, malaria symptoms, previous history of HIV infection, laboratory examinations, and outcome status (survival or death) was anonymously retrieved from individual medical records. HIV infection was previously determined by two positive rapid diagnostic tests (RDTs) and confirmed by an immunoassay test, as defined by the Brazil Ministry of Health [31]. Diagnosis was extracted from the EMR system. *Plasmodium vivax* infection was confirmed by a positive thick blood smear, as defined by the Brazil Ministry of Health guidelines [30], and diagnostic information was subsequently recorded in the hospital’s EMR and/or the Malaria Epidemiological Surveillance Information System (SIVEP-Malaria) [31]. Patients were classified with severe malaria according to the World Health Organization (WHO) guidelines [32].

### Systematic review

A systematic review regarding studies of HIV/PvCo was conducted in conformity with the Preferred Reporting Items for Systematic Reviews and Meta-Analyses (PRISMA) guidelines [33, 34]. Studies reporting HIV/PvCo were systematically identified from multiple electronic databases (Medline/PubMed, Lilacs and Scielo), using the following keywords as the search strategy: (HIV AND malaria) OR (AIDS AND malaria) OR (HIV AND *vivax*) OR (AIDS AND *vivax*) OR (HIV AND *Plasmodium*) OR (AIDS AND *Plasmodium*). The last search was performed in February 2020. No date or language restrictions were applied. All types of study design, with primary clinical data, were included (cross-sectional, longitudinal, case reports, and case series). All duplicates were removed. Additional studies were obtained by an additional search of the references from the included studies.

Study titles and abstracts were reviewed to confirm the inclusion of data on HIV/PvCo and *P. vivax* mono-infections. Included studies were assessed for eligibility by a full-text review and excluded when an inconclusive diagnosis of *Plasmodium *species and/or a doubtful HIV-positive co-infection was reported. Two independent authors of the study conducted the systematic review process. Disagreements were resolved by consensus.

For cross-sectional and longitudinal studies, the following data were retrieved: author, year of publication, country, total of malaria cases, total of vivax malaria cases, vivax malaria cases with prior HIV, mean age of population, co-morbidities and co-infections, and clinical outcomes. From the reports and case series, demographic, clinical and laboratory data were retrieved. Baseline patient characteristics were summarized as medians, with interquartile range (IQR) or means with standard deviation (SD).

### Ethical considerations

The FMT-HVD Ethics Review Board approved this study as per the guidelines and standards for regulating research on human subjects established in Resolution 466/12, of the National Health Council of the Brazilian Ministry of Health. A waiver of informed consent was obtained due to the retrospective nature of the study. Patient anonymity was preserved throughout data extraction and analysis.

## Results

### Case series

**Fig. 1:**
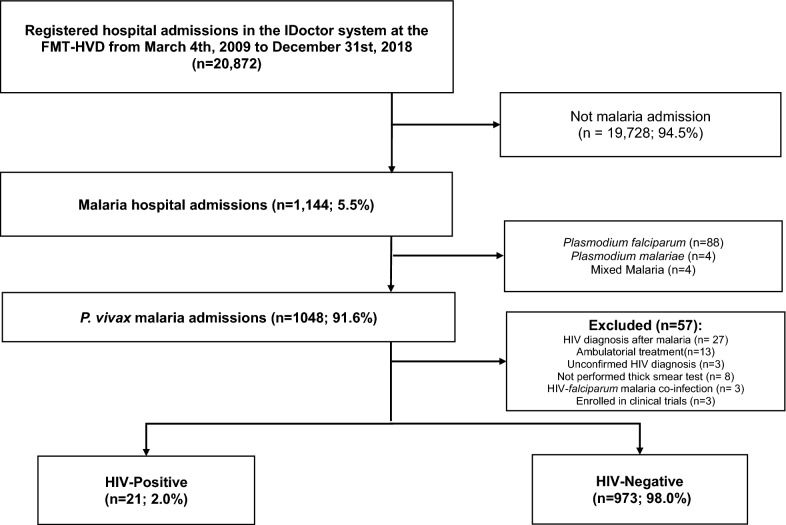
Study flowchart of hospitalized HIV-positive (cases) with *Plasmodium vivax* malaria

A total of 1,144 patients (5.5% of all FMT-HVD hospitalizations) were admitted with a malaria diagnosis during the study period. Of these, 1,048 (91.6%) were diagnosed with *P. vivax* mono-infection, out of which 21 (2.0%) were also HIV-positive (Fig. 1). Nineteen patients (90.6%) resided in Manaus’ urban area, one (4.7%) in the peri-urban area and one (4.7%) in Presidente Figueiredo, a municipality 107 km north of Manaus. None of the patients was declared as indigenous.

Table 1 shows a summary of HIV/PvCo patients. Fourteen (66.7%) were men. The mean age was 33 (± 14.2 years), with the youngest patient being a 14-year-old boy (case 6). According to HIV status, 12 (57.1%) patients were living with HIV; 9 (42.9%) presented an AIDS-defining disease; 7 (33.3%) were diagnosed with HIV at hospital admission. Prior HIV viral load and CD4 + T cell counts were reported in 12 (57.1%) patients, with an average of 32.188 (± 74,514) copies/mL and 386 (± 306.4) cells/μL, respectively. A CD4 + T cell count of < 200 cells/μL was recorded in 8 (42.1%) patients. The use of anti-retroviral treatment (ART) was registered in 14 (66.7%) patients; however, only 3 (14.3%) showed adherence to treatment in the 6 months before hospital admission.

**Table 1:** Clinical and laboratorial characteristics of Pv/HIV Co hospitalized patients at FMT-HVD

ID	Sex/age	Viral load (copies/mL)	CD4 + cell count (cells/μL)	First malaria episode/semiquantitative Parasitemia (+)^c^	Concomitant conditions/co-morbidities	AIDS defining illness	ARV treatment/adherence	Severe malaria (WHO criteria)
1	F, 22	19,109	130	Yes/+ +	Pregnancy (22 weeks)	No	No	No
2	M, 51	0	1202	No/+ +	None	No	No	No
3	F, 27	ND	6^b^	No/+	OC, TB (5th month treatment)	Yes	3Tc + AZT + ATV/r Low adherence.	No
4	M, 52	ND	ND	Yes/+ +	None	No	3Tc + AZT + LPV/r. Low adherence	No
5	F, 24	1,740	536	No/+ +	G6PD*d*	No	No	Significant bleeding (persistent brownish metrorrhagia) and respiratory distress
6	M, 14	162	325	Yes/+ +	None	No	AZT + 3Tc + LPV/r + RAL/ Low adherence.	No
7	M, 42	ND	ND	No/+ +	TB (2nd month treatment), NTX, SYP	Yes	TDF + 3Tc + EFV/ Low adherence	No
8	M, 27	58,825	17	No/+ +	TB, NTX, PNM, OC	Yes	AZT + 3Tc + LPV/r/ Low adherence.	No
9	M, 31	21,271	126	Yes/+ +	None	No	3Tc + AZT + EFV/yes	No
10	M, 29	90,336^b^	23^b^	No/+ + +	AIDS wasting syndrome, GTB, SYP	Yes	No	Anemia (6.7 g/dL), pulmonary edema and respiratory distress
11	M, 66	0	542	No/1/2+	None	No	3Tc + AZT + RTV/ Low adherence.	No
12	M, 26	14,763	228	Yes/+ +	TB (2nd month treatment)	No	3Tc + AZT + EFV/ Started treatment at hospital.	No
13	M, 29	6894	266	Yes/+ + +	Asthma	No	3Tc + AZT + LPV/r/ Low adherence.	No
14	F, 31	660	308	No/+ +	G6PD*d*	No	3Tc + ATV + TDF/ Yes	Hyperbilirubinemia (total bilirubin = 12.09 mg/dL) + AST (81 IU/L); ALT (105 IU/L), GGT (108 IU/L), LDH (609 IU/L)
15	F, 19	88,561^b^	195^b^	Yes/+	NTX	Yes	No	No
16	F, 49	56^b^	188^b^	Yes/+	Hypertension, DM, Obesity, HCV, H*er*Z, AKI	Yes	3Tc + TDF + DTG/ Yes	No
17	M, 13	230	417	Yes/1/2 +	None	No	3Tc + AZT + LPV/r Low adherence.	No
18	M, 51	ND^a^	ND^a^	No/+ +	NTX	Yes	AZT + 3TC + LPV/r Low adherence.	No
19	M, 50	262,604	427	No/+	TB, AKI, DM, LLC, ISSO, SAL	Yes	No	Respiratory distress
20	M, 39	0^b^	145^b^	Yes/+ + +	None	No	AZT + 3TC + LPV/r Low adherence.	No
21	F, 35	ND^a^	ND^a^	Yes/+ +	TB, HIV associated wasting syndrome	Yes	HIV status unknown until hospitalization. No treatment.	Hyperbilirubinemia (5.71 mg/dL) + AST (558 IU/L) and GGT (441 IU/L) associated to other organ dysfunction, AKI (creatinine 4.5 mg/dL), metabolic acidosis and respiratory distress

M male, *F* female, *DM *diabetes mellitus, *TB* tuberculosis, *ISSO* isosporiasis, *SAL* Salmonella, *NTX* neurotoxoplasmosis, *CMV* cytomegalovirus, *ASC* *Ascaris lumbricoides*, *GIA* *Giardia *spp., *SAL* *Salmonella *spp., *SYP* Syphilis, *PNM* pneumonia, *OC* oropharyngeal infection by *Candida *spp., *LLC* lower limb cellulitis, *GTB* ganglionar tuberculosis, *PFP* peripheral facial nerve paralysis, *BC* Bowen’s disease, *HCV* hepatitis C virus, *HerZ* Herpes zoster virus, *HSV* Herpes simplex virus, *AKI* acute kidney insufficiency, *G6PDd* glucose-6 phosphate dehydrogenase enzyme deficiency, *3Tc* lamivudine, *AZT* zidovudine/azidothymidine, *ATV/r* atazanavir/ritonavir, *TDF* tenofovir, *EFV* efavirenz, *DTG* dolutegravir, *LPV/r* lopinavir/ritonavir, *RAL* raltegravir, *ND* not defined

^a^RDT positive at hospital admission

^b^At hospital admission

^c^1/2 + (200–300 parasites/mm^3^);  + (301–500 parasites/mm^3^); +  + (501–10,000 parasites/mm^3^); +  +  + (10,001– 100,000 parasites/mm^3^); and +  +  +  + (> 100,001 parasites/mm^3^)

Of the included patients, 11 (52.4%) reported a first malaria episode. A previous history of co-morbidities and co-infections was present in 14 (66.7%) patients. Of these, 7 (33.3%) reported a diagnosis of HIV, vivax malaria and tuberculosis (TB) co-infection. One (4.8%) female patient was pregnant. She was treated solely with chloroquine for 3 days. According to WHO guidelines [35], severe malaria criteria (significant bleeding, respiratory distress, severe anaemia, pulmonary oedema, metabolic acidosis, and hyperbilirubinaemia, associated with organ dysfunction) were present in 5 (23.8%) patients (cases 5, 10, 14, 19, and 21) (Table 1).

One (4.8%) patient with severe malaria, who was treated with intravenous artesunate, died on the day following hospitalization (case 21). Two (9.5%) patients (cases 5 and 14) returned to the hospital after discharge with signs of haemolysis and were diagnosed with G6PD deficiency (G6PDd). In both cases, treatment with primaquine was stopped. The remaining patients completed anti-malarial treatment with chloroquine and primaquine. Malaria treatment was performed according to the Brazilian Ministry of Health guidelines [30].

Anaemia (haemoglobin level < 12 g/dL) was present in 16 (76.2%) patients, one of whom was severely anaemic (Hb < 7 g/dL). Low platelet levels (< 150,000/cu mm) were recorded in 18 (85.7%) patients, while severe thrombocytopenia (< 50,000/cu mm) was observed in 10 (47.6%) patients, with a single subject presenting significant bleeding (case 5) (Table 1).

### Systematic review

**Fig. 2:**
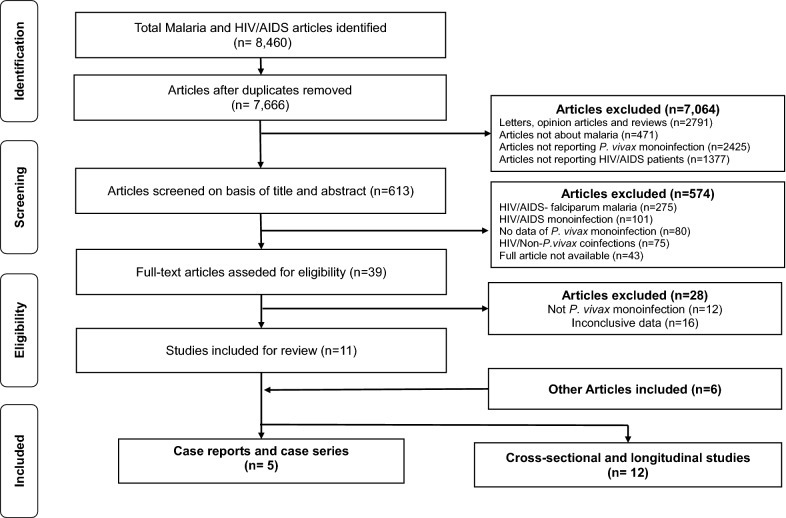
Flow chart of inclusion of studies reporting HIV- *Plasmodium vivax* co-infection

The original search yielded 8,460 studies. After the exclusion of duplicates, screening and the use of predefined inclusion criteria, only 11 studies were included for further analysis (Fig. 2). Subsequently, 6 other studies were added after a reference search of the included studies. The selected studies were reviewed and thereafter stratified into 2 main groups; the first group comprised 12 cross-sectional or longitudinal studies (Table 2) and the second group was composed of 5 case report studies (Table 3).

**Table 2:** HIV-*Plasmodium vivax* co-infections according by country

Author, year	Country (city)	Type of study/ study population	Total malaria cases	Total *P. vivax* malaria cases	Vivax malaria cases with prior HIV/AIDS	Mean age of population (± SD)/(IQR)
Volsky, 1986 [43]	Venezuela (Tachira)	Cross-sectional/patients presenting to hospital for malaria diagnosis	24	12	5^a^ (20.8)	10–60
Lo, 1991 [44]	Brazil (São Paulo)	Cross-sectional/selected patients seeking care for malaria, who had shared injectable drugs. HIV and vivax malaria were transmitted by needle sharing in most cases	12	12	3 (25.0)	24
Barata, 1993 [45]	Brazil (São Paulo)	Cross-sectional/selected patients seeking care for malaria, who had shared injectable drugs. HIV and vivax malaria were transmitted by needle sharing needle sharing in most cases	99	24	Unknown 99 tests, 24 *P. vivax* malaria, 52 HIV +	23 (± 6.3)
Erhabor, 2006 [46]	Nigeria (Niger Delta)	Case–control within an ART program/ patients attending a health facility	30	2	2 (6.7)	35.2 (± 1.29)
Ramírez-Olivencia, 2011 [83]	Spain (Madrid)	Retrospective case-series/patients diagnosed with malaria in a local hospital	398	8	1 (0.25)	36.5 (31–47)
Bharti, 2012 [47]	India (Chennai)	Cross-sectional/subjects randomly selected from newly diagnosed HIV-1 + individuals seen at a Voluntary Counseling and Testing Center	45	27	27 (60.0)^b^	40(± 9)
Wondimeneh, 2013 [48]	Ethiopia (Gondar)	Retrospective/HIV + adult individuals with febrile illness	73	20	20 (27.4)^b^	33.5 + 9
Douglas, 2014 [49]	Indonesia (Papua province)	Retrospective/all *P. vivax* individuals attending to a hospital	3495	3495	5 (0.1)	3.1 (1.8–24.5)
Rattanapunya, 2015 [50]	Thailand (Tak province)	Cross-sectional/malaria patients attending to clinic	867	350	9(1.0)	ND
Mohapatra, 2017 [51]	India (Manipur and Mizoram)	Prospective/ follow-up of HIV + individuals	333	22	3(0.9)^b^	28.9 (± 6.3) (Manipur); 34.5 (± 6.5) (Mizoram)
Sahle, 2017 [52]	Ethiopia (Ethiopia)	Cross-sectional/HIV + adults	86	3	3 (3.5)^b^	31.95 (± 7.6)
Wondimeneh, 2018 [53]	Ethiopia (Kolla-Diba)	Cross-sectional/febrile patients attending to hospital	91	35	4(11.4)	28 (± 15.7) males; 28 (± 14.7) females

*SD* standard deviation, *ND* not defined, *ART* antiretroviral therapy

^a^ Recently HIV diagnosed

^b^Study including only HIV + patients

**Table 3:** HIV-*Plasmodium vivax* co-infection case reports

Author, year	Sex/age	Viral load (copies/mL)	CD4 + cell count (cells/μL)	Primoinfection/semiquantitative parasitemia (+)	Concomitant conditions/co-morbidities	AIDS defining illness	ARV treatment/adherence	Severe malaria (WHO criteria)
Katongole-Mbidde, 1988 [36]	F, 37	ND^a^	ND^a^	ND/ND	PNM (*Pneumocystis jirovecii*)	Yes	ND	No
McIver, 2010 [37]	M, 57	0	500	ND/+ + +	PNM	Yes	3TC + AZT + ATV/yes	No
Tano, 2014 [84]	M, 50	2352	115	No/patient was diagnosed by IFI	ND	No	ND/No	Anemia (5.6 g/dL)
Ranaweera, 2018 [38]	M, 36	ND	ND	No/+ + + ^b^	TB/PNM (*Pneumocystis jirovecii*)	Yes	ND	Hyperbilirubinemia (total bilirubin = 5.03), Shock (Systolic BP < 80 mmHg)
Montenegro-Idrogo, 2019 [39]	F, 35	105,000^b^	350^b^	Yes/+ +	ND	No	No	Impaired consciousness (Glascow coma score 6/15)
	M, 43	ND	ND	Yes/+ ^b^	ND	No	No	No

*M* male, *F* female, *BP* blood pressure, *TB* tuberculosis, *PNM* pneumonia, *3Tc* lamivudine, *IFI* indirect immunofluorescence, *AZT* zidovudine/azidothymidine, *ATV* atazanavir, *TDF* tenofovir, *ND* not defined

^a^RDT positive at hospital admission

^b^At hospital admission

The highest prevalence of studies reporting HIV/PvCo was found in the African and Asian regions, with 35.3 and 29.4%, respectively. The prevalence of reported co-infections ranged from 0.1 to 60%; patient ages ranged from 10 to 60 years. No cases of severe malaria, according to WHO guidelines [32], were reported. Data from the 6 case reports are presented in Table 3. The mean age of these patients was 51.6 (± 13.6 years). Prior and recent HIV viral load and CD4 T-cell count tests were reported in 3 (50.0%) patients and the use of antiretroviral therapy (ART) was described in 2 (33.3%) cases, with one patient adhering to treatment. *Pneumocystis* sp. pneumonia and TB were reported in three cases (50.0%) [36, 37]. Only 2 (33.3%) patients presented severe malaria, according to WHO guidelines [38, 39].

## Discussion

Both malaria and HIV are highly prevalent in tropical and sub-tropical regions of the world, which may result in an increased prevalence of such co-infection [40]. Several studies have reported on HIV and *P. falciparum* co-infection, mainly in Africa [40–42], but only a few studies have described cases of HIV/PvCo patients. Indeed, from the initial search, only 2% of studies dealt with HIV/PvCo. This could be due to several factors, such as low co-infection rates, low prevalence of severe cases and therefore lack of reporting, and most importantly, lack of systematic HIV screening in vivax malaria-positive patients.

HIV and malaria affect millions of people in overlapping geographical areas. Traditional risk factors for HIV and vivax malaria may apparently be dissociated, which could possibly explain the HIV/PvCo low prevalence reported here compared to similar studies from other vivax endemic regions [43–53]. Local characteristics may account for the predisposition of HIV/PvCo. About 90% of all malaria episodes in Brazil is caused by *P. vivax* and these are concentrated in the Amazon region [54]. The clinical spectrum varies from asymptomatic cases to mild clinical symptomatology, and complications may occur. Male subjects present a higher incidence of malaria with younger individuals predisposed to a higher risk of clinical complications [55]. Malaria transmission in the Amazon region occurs mainly in peri-urban and rural areas, with an increase in the number of cases occurring in the dry season and after public holidays and regional festivities [56]. On the other hand, HIV infection, which is prevalent in 0.4% of the general population in Brazil, occurs mainly in key groups, such as in men who have sex with men, sex workers and transgender individuals [57]. For instance, in 2019, the AIDS detection rate was 29.1 cases/100,000 habitants in the state of Amazonas, while in the capital, Manaus, 46.9 cases/100,000 habitants, which is significantly higher when compared to the rest of the state, and in comparison with the Brazilian national rate (17.8 cases/100,000 habitants) [57]. HIV transmission occurs mainly in urban environments [58]. In the state of Amazonas, Manaus, Parintins and Tabatinga cities are some of the major HIV hotspots [59]. In most municipalities in the interior of the state of Amazonas, there is not a clear delimitation between urban, peri-urban and rural regions. Constant population displacement between such regions and between municipalities is very common. This may partially explain the exposure of people living with HIV to malaria. In addition, it is uncertain to what extent cultural and social characteristics significantly overlap between both diseases to produce a low prevalence as seen in this study. However, since the systematic screening of HIV infection is not done in acute malaria cases in Brazil, the real HIV/PvCo burden is unknown. Furthermore, it is important to mention that HIV-positive cases have been increasing in recent years in the northern region of Brazil [57].

Separately, HIV, malaria and TB are considered to be the most common and severe infectious diseases in the world [60]. Interestingly, a triple infection with these three diseases was more prevalent in this study when compared with other studies from Africa, although this may be attributed to screening, as previously mentioned [42, 61].

Two patients presented with G6PDd. The lower the G6PD activity, the lower the individual’s ability to tolerate oxidative stress and, when faced with oxidative stressors, such as certain foods, e.g., fava beans and drugs, such as primaquine or sulfonamides, G6PDd individuals may develop acute life-threatening haemolysis [62]. The prevalence of G6PDd applies to both HIV and malaria. HIV infection and antiretroviral therapy (ART) are both associated, separately and together, with increased oxidative stress. The impact of G6PDd on the oxidative stress of people living with HIV (PLHIV) on ART is controversial [63–65]. PLHIV have significantly lower levels of antioxidants, haematological parameters and CD4 + T cells compared to healthy subjects. Nonetheless, PLHIV on ART have presented a higher level of antioxidants compared to ART naïve subjects [67]. Also, antioxidant status has been shown to be significantly higher in those with CD4 + T cells ≥ 200/cu mm [66].

The prevalence of G6PDd varies across Latin American and Caribbean countries, with the African variant present in a wide range in this region [67]. Primaquine is a strong oxidative drug and may cause severe acute haemolysis in G6PDd individuals who take primaquine for malaria treatment [68]. G6PDd testing is currently recommended by WHO prior to starting primaquine in the radical cure of *P. vivax* and *Plasmodium ovale* malaria [30, 69]. Systematic screening for G6PDd is however not a requirement when starting HIV treatment [29]. Therefore, it was not possible to determine whether G6PD deficiency in HIV/PvCo influenced clinical outcomes.

Separately, malaria and HIV cause significant laboratory abnormalities; a co-infection scenario may intensify such alterations [27, 40]. Anaemia, thrombocytopenia, and leukopaenia, for both malaria and HIV, and in malaria/HIV co-infected patients, have been reported to be strong and independent predictors of morbidity and mortality [52]. The prevalence of anaemia in this case series was high, with most patients presenting mild to moderate anaemia, similar to other studies of HIV/PfCo [26, 70, 71]. Nonetheless, this is higher when compared to *P. vivax* mono-infected adults from the Amazonas [72].

A high prevalence of thrombocytopenia (85.7%) was also observed in this study. Two studies conducted in patients with *P. vivax* showed a similar prevalence (62.9 and 72%, respectively) [73]. In a systematic review [74], severe and fatal thrombocytopenia was observed in 10.1% of patients with vivax mono-infection malaria, while severe thrombocytopenia was more prevalent in this study. Anaemia and thrombocytopenia in HIV-malaria co-infections have a multifactorial origin and are a frequent complication that may become clinically important in HIV infection [40, 52, 75].

The impact of HIV on the clinical severity of falciparum malaria seems to be primarily motivated by the inability of the immune system to control the parasitic burden [41, 76, 77]. Severe malaria was observed in approximately 30% of adults with falciparum malaria and HIV in the urban area of Burkina Faso [78]. Studies in areas of low malaria transmission in South Africa and India showed an association between severe malaria and HIV [76, 79–81]. For severe vivax malaria, the current study showed a higher prevalence compared to another study with severe vivax malaria in children and adult patients (23.8 vs 12.6%) [82]. Despite the low number of cases, HIV co-infection seems to exacerbate clinical worsening of vivax malaria, as it has a higher prevalence than that found in vivax malaria mono-infection patients treated at FMT-HVD (~ 14%) [55]. Some studies have shown that the risk of malaria severity increases in HIV patients with a CD4 + T cell count < 200 × 10^6^ cells/L or < 350 cells/μL [14, 80]. In this study, 42.1% of patients with malaria infection had CD4 + T cell counts of less than 200 cells/μL, and one of them had severe malaria.

This study had several limitations. Regarding the systematic review, prevalence studies and those exploring severe clinical outcomes may underestimate the HIV/malaria co-infection, since it is rarely screened in vivax malaria-endemic regions. The comparison of clinical disease dynamics and important outcomes was not possible due to the absence of control groups, e.g., *P. vivax* mono-infection and HIV mono-infection to address associations of laboratory and clinical outcomes with HIV/PvCo, which was mainly due to the lack of systematic screening. Moreover, there is a low prevalence of severe cases of *P. vivax*, especially when opportunely diagnosed and treated, or in the absence of co-morbidities. Despite the analysis of the present results, it is not possible to assume that malaria increases anaemia and thrombocytopenia in PLHIV, or *vice-versa*. Finally, an accurate prevalence of HIV/PvCo, and roughly all other co-infections, is significantly hampered by the absence of a systematic screening, which in low- and middle-income countries is performed at the discretion of a clinician upon clinical suspicion.

## Conclusion

Malaria from *P. vivax* infection appears to have a low prevalence in HIV-infected individuals, with only a few studies describing clinical and laboratory outcomes. This study showed a low prevalence of HIV/PvCo, despite the important local prevalence of vivax malaria and HIV diseases separately. Even though relatively small, thus far this is the largest case series to describe HIV/PvCo. The findings described here shows the need for further research on the interaction between HIV and vivax malaria diseases, due to the potential worsening of both disease conditions in a co-infection scenario, the impact of G6PDd, and the possible epidemiological effects. At present, with a potential increase of severe cases due to the increase of vivax malaria cases and new HIV diagnoses, prospective studies are needed to elucidate aspects related to the pathogenesis of this co-infection, concomitant treatment and drug interactions, severity outcomes, and the possible increased frequency of *P. vivax* relapses secondary to HIV co-infection. In addition, future studies should address how vivax malaria chronically influences CD4 cells and how this is associated with HIV viral load dynamics.

## Abbreviations

AIDS: Acquired immunodeficiency syndrome
ART: Antiretroviral treatment for HIV/AIDS
CD4: CD4 + T-cells
EMR: Electronic medical record
G6PD: Glucose-6-phosphate dehydrogenase
FMT-HVD: Fundação de Medicina Tropical Dr. Heitor Vieira Dourado
HIV: Human immunodeficiency virus
HIV/PfCo: HIV-*Plasmodium falciparum* co-infection
HIV/PvCo: HIV-*Plasmodium vivax* co-infection
PRISMA: Preferred reporting items for systematic reviews and meta-analyses
RDT: Rapid diagnostic tests
SIVEP: National epidemiological surveillance system for malaria
TB: Tuberculosis
WHO: World Health Organization

## Availability of data and material

All data generated from this study are included in the manuscript.
